# Interest in and Use of Smoking Cessation Support Across Pregnancy and Postpartum

**DOI:** 10.1093/ntr/ntz151

**Published:** 2019-08-23

**Authors:** Felix Naughton, Luis Reeves Vaz, Tim Coleman, Sophie Orton, Katharine Bowker, Jo Leonardi-Bee, Sue Cooper, Laura Vanderbloemen, Stephen Sutton, Michael Ussher

**Affiliations:** 1 School of Health Sciences, University of East Anglia, Norwich, NR4 7TJ, UK; 2 Division of Primary Care, UK Centre for Tobacco and Alcohol Studies and National Institute for Health Research School for Primary Care Research, University of Nottingham, Nottingham, UK; 3 Division of Epidemiology and Public Health, University of Nottingham, Nottingham, UK; 4 Department of Primary Care and Public Health, Imperial College London, London, UK; 5 Behavioural Science Group, Institute of Public Health, University of Cambridge, CB2 0SR, UK; 6 Population Health Research Institute, St Georges, University of London, London, UK; 7 Institute for Social Marketing and Health, University of Stirling, Stirling, UK

## Abstract

**Background:**

Limited research exists on interest in and use of smoking cessation support in pregnancy and postpartum.

**Methods:**

A longitudinal cohort of pregnant smokers and recent ex-smokers were recruited in Nottinghamshire, United Kingdom (*N* = 850). Data were collected at 8–26 weeks gestation, 34–36 weeks gestation, and 3 months postpartum and used as three cross-sectional surveys. Interest and use of cessation support and belief and behavior measures were collected at all waves. Key data were adjusted for nonresponse and analyzed descriptively, and multiple regression was used to identify associations.

**Results:**

In early and late pregnancy, 44% (95% CI 40% to 48%) and 43% (95% CI 37% to 49%) of smokers, respectively, were interested in cessation support with 33% (95% CI 27% to 39%) interested postpartum. In early pregnancy, 43% of smokers reported discussing cessation with a midwife and, in late pregnancy, 27% did so. Over one-third (38%) did not report discussing quitting with a health professional during pregnancy. Twenty-seven percent of smokers reported using any National Health Service (NHS) cessation support and 12% accessed NHS Stop Smoking Services during pregnancy. Lower quitting confidence (self-efficacy), higher confidence in stopping with support, higher quitting motivation, and higher age were associated with higher interest in support (*p*s ≤ .001). A recent quit attempt and greater interest in support was associated with speaking to a health professional about quitting and use of NHS cessation support (*p*s ≤ .001).

**Conclusions:**

When asked in early or late pregnancy, about half of pregnant smokers were interested in cessation support, though most did not engage. Cessation support should be offered throughout pregnancy and after delivery.

**Implications:**

There is relatively high interest in cessation support in early and late pregnancy and postpartum among smokers; however, a much smaller proportion of pregnant or postpartum women access any cessation support, highlighting a gap between interest and engagement. Reflecting women’s interest, offers of cessation support should be provided throughout pregnancy and after delivery. Increasing motivation to quit and confidence in quitting with assistance may enhance interest in support, and promoting the discussion of stopping smoking between women and health practitioners may contribute to higher support engagement rates.

## Introduction

Reducing smoking rates in pregnancy remains a global public health priority.^1^ While smoking in pregnancy rates have continued to reduce over the last decade,^2^ rates remain relatively high in many European and American nations. The United Kingdom is among the countries with the highest smoking in pregnancy rates.^2^ Based on routinely collected data at the time of delivery in England in 2018,^3^ recent reductions of smoking in pregnancy rates appears to have stalled, remaining at 11%. Increased efforts and new approaches are likely needed in order to reach the English national ambition of no more than 6% of women smoking in pregnancy by the end of 2022.^4^

One key approach to reducing smoking in pregnancy is the provision of smoking cessation support. Guidance from the UK National Institute for Health and Care Excellence (NICE) recommends that all pregnant women are carbon monoxide (CO) breath tested, with all those identified as smokers provided with risk information and referred to local National Health Service (NHS) Stop Smoking Services (SSS) for specialist behavioral support and nicotine replacement therapy (NRT) where appropriate.^5^ This “opt-out” referral pathway can increase both access rates to the English SSS, from 11% to 18% in one site,^6^ and abstinence rates.^6,7^ Use of other smoking cessation support among pregnant smokers is largely unknown. This includes discussions about stopping smoking with health professionals, the use of the NHS telephone helpline, and use of NRT outside of the SSS other than in primary care, where NRT prescription rates around the time of pregnancy are estimated to be 11%.^8^

A likely important factor in accessing or accepting the offer of cessation support in pregnancy is an individual’s interest in the support being offered. There have been very few assessments of interest in cessation support among pregnant smokers and, when undertaken, this has been only for a limited selection of interventions. An English cross-sectional study conducted in 2004 found that 60% of pregnant smokers interviewed in early pregnancy indicated an interest in receiving help with stopping, with interest highest for in-person behavioral support followed closely by self-help materials.^9^ We are not aware of any longitudinal studies examining interest in cessation support over time during pregnancy, nor for a wider selection of intervention types, though findings have shown that quit attempts continue throughout pregnancy and postpartum, suggesting interest may be maintained over time.^10^ Findings from such work could help prioritize which type of support to offer and when. For example, in recent years, self-help has emerged as a promising and low-cost approach to supporting cessation in pregnancy. Reviews have demonstrated that self-help^11^ and digital self-help^12^ cessation interventions are effective in pregnancy. Currently, though, we do not know how pregnant smokers view these types of interventions and how interested they are in using them.

Little is also known about which characteristics of pregnant smokers are associated with interest in or the uptake of cessation support. Existing studies have shown that interest in cessation counseling among pregnant smokers is associated with being older, having a lower income, having a significant other who advises quitting, and lower quitting confidence.^13^ Accepting a referral or accessing specialist cessation support is associated with having a mental health problem, when pregnant women with and without mental health problems were compared,^14^ and, among postpartum smokers, higher education.^15^ However, these cross-sectional studies have only investigated a relatively narrow range of potential predictors. Identifying predictors of interest and uptake of a variety of cessation support in this population will help guide and inform interventions that aim to increase support engagement.

The primary aim of this study was to assess the interest in, use of, and attitudes toward smoking cessation support during pregnancy and the immediate postpartum period among current or recent ex-smokers. Our secondary aims were to identify predictors of interest in and use of cessation support during pregnancy and to identify perceived barriers to using self-help cessation support over the pregnancy and postpartum period.

## Methods

### Design

Three cross-sectional surveys, taken from a longitudinal cohort of pregnant and postpartum women (the Pregnancy Lifestyle Survey), were used for this study.^10,16^ Data were collected at 8–26 weeks gestation (wave 1), 34–36 weeks gestation (wave 2), and 3 months after childbirth (wave 3).

### Participants

Women aged 16 years or above, between 8 and 26 weeks pregnant, and who self-reported being either current smokers (self-reported occasional smokers or daily smokers) or having smoked in the 3 months prior to becoming pregnant were eligible for participation. Women who were unable to understand study procedures sufficiently to provide consent or were unable to read or understand the written questionnaires in English were excluded.

### Procedure

Recruitment to the Pregnancy Lifestyle Survey took place between August 2011 and August 2012 at two antenatal clinics within Nottingham University Hospitals NHS Trust (City Hospital and Queen’s Medical Centre). To ensure representative sampling, researchers attended on average five different clinic sessions per week. All women self-reporting to be between 8 and 26 weeks gestation attending routine antenatal appointments at these clinics were invited by a researcher or a member of clinic staff to complete an anonymous screening questionnaire to determine study eligibility. Those who met the criteria were directed to read a participant information sheet describing the study and, if willing, to then complete a baseline questionnaire; women could also seek further information from the researcher/staff member. On completion of the baseline questionnaire, women were offered a £5 high street shopping voucher as recognition for the time taken to complete the questionnaire. Written informed consent was obtained from those who wished to complete the two follow-up questionnaires, who made up the sample of the study.

All participants were posted a second questionnaire at 34–36 weeks gestation. In addition, participants who provided an e-mail address were e-mailed a link to a web-based version of the questionnaire and sent one e-mail reminder. Nonrespondents were sent one postal/e-mail reminder letter and then contacted by telephone; if no response was received, participants were texted a reminder. Participants who were successfully contacted via telephone were invited to complete the questionnaire during the call.

Participants were sent the final questionnaire 3 months after their delivery using the same method as described above for follow-up in later pregnancy. All participants who completed follow-up questionnaires were sent a £5 shopping voucher for each follow-up.

A more detailed description of the procedure for enrollment and data collection and sample characteristics is provided elsewhere.^16^ The study was approved by Derbyshire Research Ethics Proportionate Review Sub-Committee (11/EM/0078).

### Measures

Copies of the questionnaires used at each wave can be found in a separate publication.^10^ The questions used in the current study are described below and used a range of response formats including yes/no responses, multiple choices, and five-point Likert-type scales for attitudinal items.

#### Interest, Use, and Attitudes Toward Smoking Cessation Support

At all three waves, participants were asked to rate their interest in receiving help with stopping smoking in general and their interest in, difficulty in using, and perceived usefulness of nine different types of smoking cessation support using a 1–5 scale (“not at all” to “extremely”). These were split into health practitioner-orientated support (telephone helpline, group sessions, and one-to-one sessions) and self-help support (booklet, DVD, web site, text messages, e-mail, and smartphone/digital device application). Participants were also asked to indicate whether they had accessed any of the cessation support offered by the NHS, though not necessarily delivered by the NHS, since finding out they were pregnant (wave 1) or since completing the last survey (waves 2 and 3). These included talking to a General Practitioner (GP)/nurse or midwife about stopping smoking, attending an NHS Stop Smoking Service group or individual session, calling a stop smoking helpline, or using NRT (from any source). At baseline only, participants were asked about their access to electronic/digital devices for using self-help. In all three waves, participants were asked to indicate from a list of statements, informed by prior work,^13^ any potential barriers toward accessing or using self-help cessation support.

#### Predetermined Predictor Variables of Interest and Use of Cessation Support

A broad range of potential demographic and psychosocial predictors were included based on previous evidence in predicting interest or uptake in cessation support^13–15^ and cessation^17,18^; although relatively little is known about this, we had no prior hypotheses regarding potential associations. Background and health predictors included gestation, general health (general health rating from excellent to poor and whether they had a long-standing physical or mental illness or disability),^19^ depression (during the past month bothered by feeling down, depressed, or hopeless or having little interest or pleasure in doing things),^20^ the Perceived Stress Scale 4 (PSS-4),^21^ ethnicity, age, and index of multiple deprivation.^22^ Smoking-related predictors included general smoking behavior,^23,24^ urges to smoke,^25^ partner’s smoking status, nicotine dependence using the “Heaviness of Smoking Index,” ^26^ intentions and determination to quit smoking and confidence (self-efficacy) in achieving this,^27^ support for stopping smoking from friends/family, beliefs about the harm of smoking during pregnancy,^27^ and whether participant had talked to a health professional (midwife, nurse, or GP) about quitting. Smoking-related norm predictors included injunctive norm (“people important to me think I should stop smoking”) and descriptive norm (knowing others who smoked throughout pregnancy).^28^

#### Sample Size and Analysis

The sample size calculation for the cohort survey was based on estimating the number of quit attempts initiated during pregnancy among smokers and is reported elsewhere.^16^

#### Analysis

Descriptive statistics were used to report interest in and use of smoking cessation services as well as attitudes to smoking cessation support and barriers to self-help use for all three waves. For the prevalence estimates of interest in and attitudes toward smoking cessation support among smokers, responses were dichotomized by grouping the five possible responses: not at all/a little (no interest) versus moderately/very much/extremely (interest), with the same thresholds for determining low versus high perceived usefulness and difficulty accessing/using. Multiple imputation using chained equations was performed using 20 iterations^29^ for the percentage who were interested in cessation support at late pregnancy and postpartum. Missing data on interest in and attitudes toward smoking cessation support was imputed using a logit imputation method based on the following baseline factors: maternal age, ethnicity, highest educational qualification, gestation of pregnancy, smoking status, urge to smoke, general health status, depression status, parity, and smoking status in previous pregnancy.

For the exploratory analysis investigating potential correlates of general interest in cessation support at baseline, discussion of stopping smoking with a health professional at baseline, and use of NHS cessation support (telephone helpline, group sessions, one-to-one sessions, or NRT) in late pregnancy, we used linear regression and logistic regression to test the univariable associations with baseline characteristics. Interest was measured on a 1–5 scale and use was coded as a binary “use” or “nonuse” variable. Following this, all predictors that were associated with interest in or use of cessation support in the univariable analyses at *p* < .1 were included in multivariable models,^30^ providing they were not found to be collinear (variance inflation factor ≥10). Missing data for the predictor variables (i.e., not having answered survey questions at either baseline or late pregnancy follow-up) were addressed through including missing data as either an additional category for categorical variables or using a dummy variable to indicate missingness for continuous variables. As two out of three of the predictor analyses were cross-sectional and they were part of a secondary aim of the study, we did not perform multiple imputation for these.

## Results

At the late pregnancy and postpartum follow-ups, 509 (60%) and 476 (56%) of baseline participants completed a questionnaire respectively. Table 1 presents the baseline sample characteristics (*N* = 850), full details of which are reported elsewhere.^16^ On average, participants were 26 years old and 16 weeks gestation at baseline. One-third reported that the current pregnancy was their first and, among those who had been pregnant before, around two-thirds smoked during their last pregnancy. Fifty-seven percent reported being current smokers and, among these, over half had a partner who smoked. Almost all respondents owned a mobile phone (97%) and 71% percent owned a smartphone.

**Table 1.** Characteristics of the cohort (*N* = 850)

Characteristic	Mean (SD)
Gestation (weeks) (*n* = 806)	15.6 (4.1)
Age (*n* = 847)	25.8 (5.6)
Perceived stress scale (PSS-4) (*n* = 819)	10.6 (3.5)*
	*n* (%)^**^
Qualifications: GCSEs or similar^***^	594/850 (69.9)
Home ownership	166/846 (19.6)
Cars or vans available for use in household	446/839 (53.2)
In paid work	383/850 (45.1)
Ethnicity: Caucasian	783/844 (92.8)
Baseline smoker	488/850 (57.4)
First pregnancy	275/839 (32.8)
If pregnant before, smoked during last pregnancy	368/561 (65.6)
Owns a mobile phone	776/797 (97.4)
Owns a smartphone	547/774 (70.7)
Mobile phone bundle includes free text messages	610/778 (78.4)

*Maximum score on PSS-4 is 16.

^**^Numbers may not add up to 850 (total sample size) due to missing data.

^***^General Certificate of Secondary Education (GCSE) is a qualification/exam taken by school students in the United Kingdom (except Scotland) usually when aged between 14 and 16 years old.

### Interest and Use of Cessation Support and Perceived Barriers to Accessing Self-Help 

At baseline, during early pregnancy, 44% (95% CI 40% to 48%) of current smokers (Table 2) and 9% (95% CI 6% to 12%) of recent ex-smokers (not shown in table) reported being interested in receiving help with stopping smoking. Among smokers, the specific support types rated of most interest were one-to-one sessions (42%), followed by self-help booklets (39%). Imputed survey results showed that interest in support to stop smoking, among smokers, changed little in late pregnancy (43%; 95% CI 37% to 49%) relative to early pregnancy but dropped to 33% (95% CI 27% to 39%) 3 months postpartum. Self-help booklets were of most interest (49%) followed by self-help web sites (45%) in late pregnancy. Across all timepoints, one-to-one sessions had the highest perceived usefulness and self-help support had the lowest levels of perceived difficulty to access and use.

**Table 2.** Interest in and attitudes toward smoking cessation support among smokers (*N* = 488)

Type of support*	Early pregnancy (8–26 weeks gestation)	Late pregnancy (34–36 weeks gestation)^**^	Postpartum (3 months)^**^
	*n* (%, 95% CI)	% (95% CI)	% (95% CI)
Interest in receiving help with stopping	212 (44.0, 39.5 to 48.4)	42.8 (37.0 to 48.6)	32.9 (26.9 to 38.8)
Health professional telephone helpline			
Interest	92 (20.5)	25.2	21.8
Perceived usefulness	112 (24.7)	24.6	25.5
Difficulty accessing/using	153 (34.3)	30.9	31.9
Health professional group sessions			
Interest	62 (14.1)	14.9	12.2
Perceived usefulness	128 (28.3)	28.0	24.9
Difficulty accessing/using	179 (40.3)	39.5	43.0
Health professional one-to-one			
Interest	190 (41.7)	43.9	38.1
Perceived usefulness	225 (49.2)	49.8	48.3
Difficulty accessing/using	155 (34.9)	33.0	34.0
Self-help booklet			
Interest	177 (39.3)	48.9	39.3
Perceived usefulness	182 (40.1)	46.0	36.6
Difficulty accessing/using	135 (30.1)	21.5	20.3
Self-help DVD			
Interest	154 (34.8)	37.9	29.0
Perceived usefulness	163 (36.1)	39.8	29.8
Difficulty accessing/using	130 (29.3)	20.5	24.8
Self-help web site			
Interest	148 (33.9)	44.7	37.0
Perceived usefulness	160 (35.5)	42.2	32.1
Difficulty accessing/using	132 (30.1)	24.2	27.7
Self-help text messages			
Interest	133 (30.5)	40.5	30.4
Perceived usefulness	138 (30.5)	40.2	30.3
Difficulty accessing/using	128 (28.8)	23.1	22.6
Self-help emails			
Interest	112 (25.7)	32.8	24.2
Perceived usefulness	124 (27.5)	32.2	24.7
Difficulty accessing/using	138 (31.0)	25.8	24.3
Self-help phone app			
Interest	146 (33.2)	40.2	36.3
Perceived usefulness	159 (35.3)	43.3	37.0
Difficulty accessing/using	136 (30.7)	25.4	22.7

*Responses were dichotomized by grouping the five possible responses: not at all/a little (no interest) versus moderately/very much/extremely (interest). The same approach was used for perceived usefulness (low vs. high perceived usefulness) and difficulty accessing/using (low vs. high difficulty).

^**^Missing data were generated using multiple imputation “by chained equations for dichotomized interest in cessation support amongst smokers in early and late pregnancy and in postpartum period.”

In early pregnancy, less than half of smokers (43%) reported having talked to a midwife about stopping smoking and fewer had spoken to a GP or nurse (27%) about this (Table 3). Between early and late pregnancy, 27% of smokers reported speaking to a midwife about stopping. Across the whole of pregnancy, over one-third (38%) of smokers did not report having talked with either a midwife, GP, or nurse about stopping smoking (not shown in table).

**Table 3.** Reported use of National Health Service (NHS) provided smoking cessation support split by smoking status

Type of support*	Early pregnancy		Early–late pregnancy^**^		Postpartum	
	Recent ex-smoker (*N* = 362)	Smoker (*N* = 488)	Recent ex-smoker (*N* = 265)^***^	Smoker (*N* = 256)^***^	Recent ex-smoker (*N* = 196)	Smoker (*N* = 280)
	*n* (%)	*n* (%)	*n* (%)	*n* (%)	*n* (%)	*n* (%)
Talked to GP/Nurse about giving up	22 (6.1)	132 (27.0)	3 (1.1)	28 (10.9)	3 (1.5)	36 (12.9)
Talked to midwife about giving up	36 (9.9)	211 (43.2)	16 (6.0)	68 (26.6)	3 (1.5)	24 (8.6)
Accessed/used any NHS smoking cessation support	29 (8.0)	90 (18.4)	21 (7.9)	66 (25.8)	6 (3.1)	38 (13.6)
Attended NHS stop smoking service group session	3 (0.8)	21 (4.3)	1 (0.4)	10 (3.9)	0 (0.0)	5 (1.8)
Attended solo/individual NHS stop smoking service session	10 (2.8)	25 (5.1)	15 (5.7)	23 (9.0)	1 (0.5)	7 (2.5)
Called a stop smoking telephone helpline	5 (1.4)	18 (3.7)	3 (1.1)	7 (2.7)	0 (0.0)	5 (1.8)
Used nicotine replacement therapy	25 (6.9)	64 (13.1)	16 (6.0)	57 (22.3)	6 (3.1)	31 (11.1)

*Noncumulative support usage. Rates in each time period do not include usage in earlier time period and represent what was reported in each questionnaire.

^**^Period between the early pregnancy questionnaire and the late questionnaire. Includes 10 women who had given birth prior to completing the 34-week questionnaire

Out of those participants who smoked at all during pregnancy, 12% reported accessing Stop Smoking Service support (group or one-to-one support) at some point in their pregnancy (not shown in table). Across the whole of pregnancy 17% of participants reported accessing at least one form of NHS-provided cessation support, which rose to 27% when restricted to those reporting smoking at both baseline and end of pregnancy (not shown in table). The most common type of support used was NRT without additional behavioral support (Table 3).

In early pregnancy, the most common barriers for using self-help cessation support was preferring to receive support from a health professional (19% smokers, 6% recent ex-smokers) and thinking self-help would not be of much help with quitting (14% smokers, 7% recent ex-smokers). These remained the two most common barriers at the two follow-up timepoints.

#### Factors Associated With Interest in Cessation Support Among Smokers in Early Pregnancy

Among smokers, 11 out of 23 prespecified variables were univariable correlates of interest in receiving cessation support in general at baseline (*p* < .05; Table 4). When these were entered into a multivariable model, the following five predictors remained statistically significant: higher determination to quit (*B* = 0.42, 95% CI 0.31 to 0.54), lower confidence in stopping until the end of pregnancy (*B* = −0.23, 95% CI −0.35 to −0.12), higher confidence in stopping with health professional support (*B* = 0.22, 95% CI 0.11 to 0.33), having spoken to a health professional about stopping (*B* = 0.77, 95% CI 0.55 to 0.98), and higher age (*B* = 0.03, 95% CI 0.01 to 0.05).

**Table 4.** Univariable and multivariable models for correlates/predictors among smokers of (1) general interest in cessation support at baseline and (2) use of National Health Service cessation support in late pregnancy

Baseline variables	Dependent variable: interest in cessation support at baseline (*N* = 482)						Dependent variable: use of cessation support in late pregnancy (*N* = 263)					
	Univariable model			Multivariable model			Univariable model			Multivariable model		
	Beta	*p*	95% CI	Beta	*p*	95% CI	OR	*p*	95% CI	OR	*p*	95% CI
Smoked in a prior pregnancy	−0.126	.336	−0.38, 0.13				0.89	.690	0.51, 1.56			
Heaviness of Smoking Index	0.039	.396	−0.05, 0.13				1.08	.486	0.87, 1.34			
Urges to smoke	0.009	.791	−0.06, 0.07				1.00	.972	0.88, 1.15			
Has tried to quit (y/n)	0.716	<.001	0.47, 0.96				3.03	.001	1.60, 5.73	2.70	.006	1.34, 5.46
Number of 24-h quit attempts	−0.022	.036	−0.04, 0.00				0.97	.465	0.89, 1.06			
Seriously planning to quit	0.491	<.001	0.40, 0.58				1.65	<.001	1.30, 2.08			
Determination to stop till baby born	0.507	<.001	0.42, 0.59	0.423	<.001	0.31, 0.54	1.71	<.001	1.32, 2.20			
Confidence to stop till baby born	0.217	<001	0.12, 0.31	−0.231	<.001	−0.35, −0.12	1.24	.049	1.00, 1.54			
Confidence to stop alone	0.026	.596	−0.07, 0.12				0.89	.339	0.71, 1.13			
Confidence to stop with health professional help	0.425	<.001	0.33, 0.52	0.216	<.001	0.11, 0.33	1.36	.008	1.08, 1.70			
Beliefs on harms of smoking to baby	0.774	<.001	0.43, 1.11				3.05	.026	1.14, 8.11			
Knows others who smoked in pregnancy	−0.035	.863	−0.43, 0.36				1.40	.484	0.54, 3.61			
People important to me think I should stop	0.605	<.001	0.33, 0.88				1.69	.132	0.85, 3.33			
Has support to stop	0.304	.025	0.04, 0.57				1.73	.104	0.89, 3.36			
Partner smokes	0.041	.760	−0.22, 0.30				0.90	.729	0.50, 1.62			
Spoken to health professional about stopping	1.212	<.001	0.99, 1.43	0.765	<.001	0.55, 0.98	3.29	<.001	1.80, 6.01			
General health	−0.206	.244	−0.55, 0.14				1.67	.283	0.66, 4.24			
Depression	0.169	.276	−0.14, 0.47				0.94	.877	0.46, 1.95			
Perceived Stress Scale	0.008	.706	−0.04, 0.05				0.99	.871	0.90, 1.10			
Gestation (weeks)	−0.022	.152	−0.05, 0.01				0.97	.386	0.91, 1.04			
Ethnic minority	−0.143	.609	−0.69, 0.41				0.76	.640	0.24, 2.39			
Age	0.032	.006	0.01, 0.05	0.030	.001	0.01, 0.05	1.09	.001	1.03, 1.14	1.08	.006	1.02, 1.15
Deprivation (Index of Multiple Deprivation)	−0.004	.274	−0.01, 0.00				1.00	.638	0.98, 1.01			
Interest in support at baseline	—	—	—	—	—	—	1.91	<.001	1.53, 2.37	1.80	<.001	1.43, 2.27

#### Factors Associated With Discussing Stopping Smoking With a Health Professional Among Smokers in Early Pregnancy

Ten out of 23 prespecified baseline variables were univariable correlates (*p* < .05) of whether or not smokers reported having had a discussion with a health professional (midwife, nurse, or GP) about quitting smoking in early pregnancy (Supplementary Table S1). Of these, two remained statistically significant in the multivariable analysis: having previously tried to quit during the current pregnancy (odds ratio [OR] 3.0, 95% CI 2.0 to 4.6) and interest in support (OR 5.4, 95% CI 3.6 to 8.2).

#### Predictors of use of NHS cessation support among smokers by late pregnancy

Nine baseline variables were associated with use of NHS-provided cessation support by late pregnancy (*p* < .05) among baseline smokers (Table 4). Three variables remained statistically significant in the multivariable model: having previously tried to quit during the current pregnancy (OR 2.7, 95% CI 1.3 to 5.5), older age (OR 1.1, 95% CI 1.0 to 1.1), and interest in support (OR 1.8 95% CI 1.4 to 2.3).

## Discussion

This study found that a substantial minority of pregnant smokers are interested in getting smoking cessation support and that this level of interest is as high at the end of pregnancy and drops only a modest amount by 3 months postpartum. While there is relatively high interest in cessation support, a much smaller proportion of women accessed any cessation support in pregnancy, highlighting a gap between interest and engagement. Our study indicates that speaking to a health care professional about stopping smoking, being motivated to stop, and having low confidence in doing so without assistance could influence interest in NHS-provided cessation support. Interest in support, in turn, prospectively predicted use of NHS cessation support. However, women who had not tried to quit early on in their pregnancy were less likely to have used support later on in pregnancy compared with those who had tried to quit, independent of quitting motivation and interest in support. This suggests that the process of trying and failing to quit may be helpful for some by prompting them to seek assistance.

### Strengths and Limitations

We believe this was the first longitudinal cohort study looking at smoking cessation support interest and use over pregnancy and into the postpartum period. We examined women’s views and use of a broad range of support types, across the three timepoints in a large cohort. Although participants were recruited from one area, the sample as a whole demonstrated similar characteristics to pregnant smokers in national cohort studies^16^ and so key findings may be generalizable to pregnant smokers across the United Kingdom. Furthermore, very few prior studies have examined a wide range of potential predictors of interest and use of cessation support.

Common to many cohort studies, we experienced moderate attrition at follow-ups. Although not excessive and we minimized the impact of this by performing multiple imputation for key variables, this is a limitation. A further limitation is that we did not collect data during the second trimester of participant’s pregnancies, and so there may have been fluctuations in variables of interest that we were unable to capture. Also, we did not collect data on views and use of e-cigarettes in this survey as, when the survey was carried out, e-cigarette usage was still relatively low. We have now undertaken research to explore this in pregnancy in several separate recent studies.^31,32^ Similarly, the use of smartphone apps were somewhat lower when the study was undertaken compared to current usage, and interest in this may have grown in recent years. While our analysis investigating predictors of use of support was prospective, the correlates of interest in cessation support and discussion about stopping smoking analyses were cross-sectional and so we are limited in our ability to indicate possible directions of causality.

### Findings in Context

Our finding that just under half of pregnant smokers were interested in receiving cessation support in early pregnancy corresponds closely with Ussher et al.^9^ who found in 2004 that 60% of women were interested in cessation support in early pregnancy. As with this prior study, our data also showed similar interest levels between self-help and one-to-one cessation support, with low interest in group support. However, in our study, the point estimates for difficulty accessing one-to-one support were higher across all waves than the self-help options, suggesting perceived access could inhibit uptake, particularly in late pregnancy. No studies have previously explored the relative interest in different types of self-help among pregnant women. Booklets were of most interest across all waves and with mostly the highest perceived usefulness compared to other estimates for self-help types. Though there were only minor differences between the self-help types, e-mail appeared to be of least interest and perceived usefulness.

Despite relatively high rates of interest in one-to-one cessation support among baseline smokers (42%), only a small proportion (12%) of our cohort who smoked at all during pregnancy reported using the NHS Stop Smoking Services. This very closely matches the 11% access rate identified from NHS records in another study in the same region, prior to implementation of an opt-out pathway.^6^ Low rates of access to specialist cessation support among pregnant smokers has been reported historically^33,34^ and can be increased using an opt-out referral pathway,^6,7^, which also increases abstinence. The most common type of cessation support used by women in our cohort that the NHS provides was NRT. This is despite safety concerns and reluctance to recommend NRT among many practitioners.^35,36^ However, use of NRT without guidance or behavioral support is unlikely to be effective in pregnancy as even with guidance and support it is only of borderline efficacy^37^ and use of over-the-counter NRT is not associated with abstinence in the general population.^38^ Suboptimal adherence^39,40^ and increased nicotine metabolism^41^ indicate that higher doses of NRT combined with adherence support is likely required to make NRT more effective in pregnancy.

In line with a further study,^13^ we found higher age and lower quitting self-efficacy associated with interest in receiving support. We also found a positive association between confidence in quitting with a health professional’s help and support interest, which had not been examined before. Prior international research has found that while most practitioners working with pregnant women ask about smoking status,^42,43^ far fewer discuss cessation and only around one-third discuss treatment options.^42–44^ Our findings broadly support this from the U.K. pregnant women’s perspective – fewer than two-thirds of smokers in our sample reported discussing stopping smoking with a practitioner. U.K. research undertaken over the last two decades report similar findings suggesting that little has changed in this regard.^13,45^

### Implications for Practice

One key implication is that, based on findings related to interest levels, the offer of cessation support should be provided throughout pregnancy and postpartum. In the United Kingdom at least, currently policy relating to the offer of cessation support is weighted toward early pregnancy, even if strictly following NICE guidance.^5^ This is reflected by our findings, where we found far fewer smokers reporting a discussion with a health professional about stopping smoking in mid-to-late pregnancy compared with early pregnancy. This matches other research showing that less than one quarter of health professionals follow up women after an initial discussion about smoking,^14,44^ which can inadvertently reassure women that quitting smoking may not be a priority.^46^

To promote interest in cessation support, which was predictive of accessing support, our findings suggest that increasing motivation to stop smoking, ensuring health professionals discuss stopping smoking, and enhancing women’s confidence that their chances of stopping smoking are higher if they receive professional support are important targets for interventions. Practitioners often do not initiate discussions about quitting smoking with pregnant clients because of low levels of confidence, feelings of being underskilled, and concern about damaging the client relationship.^35^ The use of carbon monoxide monitoring may help with facilitating a discussion about stopping smoking, from both the perspective of practitioners^35,47^ and pregnant women,^48^ and enhance motivation to quit. When combined with an opt-out referral, it can increase the uptake of support.^6,7^ Training health practitioners can also increase the assessment of smoking and support provision^49^, and the use of prompts in maternity paperwork and electronic systems may also facilitate appropriate discussions.^35^ In terms of factors influencing uptake of support, being younger was associated with a reduced likelihood of using practitioner-orientated cessation support. This suggests a potential targeting opportunity.

Our findings also reinforce previous research^9^ by highlighting the popularity of self-help cessation support. Current UK guidance only recommends promoting self-help to those who accept the offer of formal cessation support but who are struggling to engage.^5^ This may be a missed opportunity, given recent evidence of effectiveness of digital self-help approaches for pregnant smokers,^12^ particularly when delivered by SMS text message.^27,50^

One less prominent but nonetheless important finding was that some of the most predictive factors in determining smoking and a failure to quit smoking in pregnancy, that is, socioeconomic status/deprivation, nicotine dependence, and having a partner that smokes,^17^ did not predict interest in or use of support in our cohort. From a public health perspective, this is positive and suggests these factors may not inhibit the seeking of and acceptance of cessation support.

### Conclusion

Almost half of pregnant smokers in our cohort were interested in help to stop smoking and this changed little from early to late pregnancy. Yet rates of discussing stopping smoking with a health professional reduced after early pregnancy and a substantial minority of pregnant smokers did not report having a discussion with a health professional about stopping at any point in pregnancy. With one-quarter of smokers accessing any NHS-provided support and less than half of these accessing stop smoking services, the gap between support interest and access indicates a missed opportunity. Our findings indicate that increasing motivation to quit, enhancing interest in support, the discussion of stopping smoking with health practitioners, and confidence in quitting with cessation support may contribute to higher support use rates. In addition, nonroutine forms of cessation support, including self-help, should be promoted given evidence of effectiveness, low cost, and their popularity among this population.

## Supplementary Material

Supplementary data are available at *Nicotine and Tobacco Research* online.

ntz151_suppl_Supplementary_Table

## Funding

This article presents independent research funded by the National Institute for Health Research (NIHR) under the Programme Grants for Applied Research programme RP-PG-0109-10020. The views expressed in this publication are those of the authors and not necessarily those of the NIHR or the Department of Health and Social Care.

## Declaration of Interests

None declared.
