# Supplementary material for: Interest in and Use of Smoking Cessation Support Across Pregnancy and Postpartum
Source: Nicotine Tob Res. 2019 Aug 23;22(7):1178–86. doi: 10.1093/ntr/ntz151 (PMC7291796; doi:10.1093/ntr/ntz151)
Supplement: ntz151_suppl_Supplementary_Table [file ntz151_suppl_supplementary_table.docx]

**Table S1:** **Univariable and multivariable models for correlates among smokers of having a discussion with a health professional about stopping smoking**

| **Baseline variables** | **DV: Discussion with a health professional about quitting (n=482)** | | | | | |
| --- | --- | --- | --- | --- | --- | --- |
|  | **Univariate model** | | | **Multivariable model** | | |
|  | **OR** | **p** | **95% CI** | **OR** | **p** | **95% CI** |
| Smoked in a prior pregnancy | 0.61 | 0.010 | 0.42,0.89 |  |  |  |
| Heaviness of Smoking Index | 0.93 | 0.278 | 0.81,1.06 |  |  |  |
| Urges to smoke | 0.99 | 0.785 | 0.90,1.08 |  |  |  |
| Has tried to quit (y/n) | 3.69 | <0.001 | 2.51,5.43 | 3.04 | <0.001 | 2.00,4.61 |
| Number of 24 hour quit attempts | 0.99 | 0.455 | 0.96,1.02 |  |  |  |
| Seriously planning to quit | 1.80 | <0.001 | 1.53,2.11 |  |  |  |
| Determination to stop till baby born | 1.81 | <0.001 | 1.54,2.13 |  |  |  |
| Confidence to stop till baby born | 1.28 | 0.001 | 1.11,1.48 |  |  |  |
| Confidence to stop alone | 1.08 | 0.275 | 0.94,1.25 |  |  |  |
| Confidence to stop with health professional help | 1.58 | <0.001 | 1.35,1.85 |  |  |  |
| Beliefs on harms of smoking to baby | 1.95 | 0.012 | 1.16,3.28 |  |  |  |
| Knows others who smoked in pregnancy | 1.03 | 0.916 | 0.58,1.84 |  |  |  |
| People important to me think I should stop | 2.45 | <0.001 | 1.60,3.74 |  |  |  |
| Has support to stop | 1.70 | 0.008 | 1.15,2.52 |  |  |  |
| Partner smokes | 1.17 | 0.426 | 0.80,1.72 |  |  |  |
| General health | 1.19 | 0.504 | 0.72,1.97 |  |  |  |
| Depression | 1.11 | 0.658 | 0.71,1.73 |  |  |  |
| Perceived Stress Scale | 1.02 | 0.529 | 0.96,1.09 |  |  |  |
| Gestation (weeks) | 0.98 | 0.398 | 0.94,1.03 |  |  |  |
| Ethnic minority | 1.17 | 0.701 | 0.52,2.64 |  |  |  |
| Age | 1.02 | 0.324 | 0.98,1.05 |  |  |  |
| Deprivation (Index of Multiple Deprivation; IMD) | 0.99 | 0.191 | 0.98,1.00 |  |  |  |
| Interest in support at baseline | 6.17 | <0.001 | 4.12,9.24 | 5.42 | <0.001 | 3.57,8.23 |
